# Selection Signatures in South African Nguni and Bonsmara Cattle Populations Reveal Genes Relating to Environmental Adaptation

**DOI:** 10.3389/fgene.2022.909012

**Published:** 2022-06-17

**Authors:** Bhaveni B. Kooverjee, Pranisha Soma, Magrieta A. Van Der Nest, Michiel M. Scholtz, Frederick W. C. Neser

**Affiliations:** ^1^ Department of Animal Breeding and Genetics, Animal Production, Agricultural Research Council, Pretoria, South Africa; ^2^ Department of Animal, Wildlife and Grassland Sciences, University of the Free State, Bloemfontein, South Africa; ^3^ Biotechnology Platform, Agricultural Research Council, Pretoria, South Africa

**Keywords:** climate change, composite, DNA methylation, genomic selection, genomic structure, indigenous, polymorphism

## Abstract

Climate change is a major influencing factor in beef production. The greenhouse gases produced from livestock production systems contribute to the overall greenhouse gas emissions. The aim of this study was to identify selection signatures within and between Nguni and Bonsmara cattle in relation to production and adaptation. For this purpose, genomic 150 K single nucleotide polymorphism data from Nguni (*n* = 231) and Bonsmara (*n* = 252) cattle in South Africa were used. Extended haplotype homozygosity (EHH) based analysis was executed within each population using integrated haplotype score (iHS). The R package rehh was used for detecting selection signatures across the two populations with cross population EHH (XP-EHH). Total of 121 regions of selection signatures were detected (*p* < 0.0001) in the Bonsmara and Nguni populations. Several genes relating to DNA methylation, heat stress, feed efficiency and nitrogen metabolism were detected within and between each population. These regions also included QTLs associated with residual feed intake, residual gain, carcass weight, stature and body weight in the Bonsmara, while QTLs associated with conception rate, shear force, tenderness score, juiciness, temperament, heat tolerance, feed efficiency and age at puberty were identified in Nguni. Based on the results of the study it is recommended that the Nguni and Bonsmara be utilized in crossbreeding programs as they have beneficial traits that may allow them to perform better in the presence of climate change. Results of this study coincide with Nguni and Bonsmara breed characteristics and performance, and furthermore support informative crossbreeding programs to enhance livestock productivity in South Africa.

## Introduction

Among different factors influencing production in the agricultural sector, the impact of climate change could be one of the most challenging (Calzadilla et al., 2014). The Intergovernmental Panel on Climate Change (IPCC) is predicting a 1.5°C increase above pre-industrial levels between 2030 and 2052 if warming continues to increase at the current rate (Intergovernmental Panel on Climate Change, 2018). Increasing occurrences of heat stress, drought and flooding events are likely, and these will have adverse effects on crop and livestock productivity over and above the impacts due to fluctuations in mean variable temperature (Intergovernmental Panel on Climate Change, 2019). For example, during the 2015 drought season 40,000 head of cattle died due to extreme heat waves experienced in the Kwa-Zulu Natal province (Willemse and Mare, 2016). The impact of climate change may be limited by using smaller framed animals, as well as using locally adapted breeds in commercial production systems (Scholtz et al., 2014). Locally adapted breeds are known for their ability to withstand changing climate conditions as well as resistance to parasites and diseases (Chan et al., 2010). The Nguni, a distinct Sanga type, is an indigenous cattle breed that displays adaptation to changing environments and is efficient in coping with various stressors such as parasites, heat spells, and lack of water availability (Mwai et al., 2015; Nyamushamba et al., 2017). Furthermore, Nguni cattle have lower metabolic energy requirements in heat challenged environments, as they regulate internal metabolic heat production with lowered feed intake (Sejian et al., 2018; Jiyana, 2019). Nguni cattle have smaller frames, with various horn shapes and hide patterns and are used for beef production. The Bonsmara is a local composite breed derived from crossing 5/8 Afrikaner (indigenous Sanga or *Bos taurus africanus*) and 3/16 Shorthorn and 3/16 Hereford (*Bos taurus*) (Strydom et al., 2001). Nguni cattle have smaller frames, with various horn shapes and hide patterns and are used for beef production. The Bonsmara is a local composite breed derived from crossing 5/8 Afrikaner (indigenous Sanga or *Bos taurus africanus*) and 3/16 Shorthorn and 3/16 Hereford (*Bos taurus*) (Strydom et al., 2001; Corbet et al., 2006). Bonsmara cattle are medium to large framed, smooth coated with tolerance to heat and ticks (Muchenje et al., 2008). The Bonsmara is known for beef production, high fertility rates and efficient performance in feedlot systems (Esterhuizen et al., 2008; Asizua et al., 2017).

Natural and artificial selection leads to changes in certain regions of the genome resulting in selection signatures that can reveal genes associated with the selected traits (Xu et al., 2015). Signatures of selection are the changes in allele frequencies, linkage disequilibrium and genetic variation over time and generate unique patterns of specific regions of the genome (Aliloo et al., 2020). Long-term selection pressures can be investigated through the detection of signatures of selection. With the utilization of advanced genetic technology, a more in depth view of the genetic status of each breed is possible. There are two main categories for identifying selection signatures. Firstly, there are signatures that can be detected within a population and secondly, signatures than can be identified across two populations (Voight et al., 2006; Tang et al., 2007). To detect selection signatures, an estimator called extended haplotype homozygosity (EHH) to search for genetic footprints of positive selection was developed by Sabeti et al. (2007). Several studies have employed this approach in composite cattle (Yurchenko et al., 2018; Singh et al., 2020) and indigenous breeds (Bahbahani et al., 2018; Mastrangelo et al., 2020). For instance, Saravanan et al. (2021) performed a scan of selection signatures in Indian taurine and indicine breeds and identified several candidate genes relating to adaptation such as heat stress (HSPA1B) and disease susceptibility (DEFB4). While, Yurchenko et al. (2018) identified signatures of selection in nine Russian cattle breeds that related to several genes such as feed intake (TMEM68, PLAG1), reproduction (HFM1, CSF2) and acclimation (RETREG1, AQP5). Interestingly, the study by Ben-Jemaa et al. (2020) identified several genes to be under positive selection in North African cattle populations, such as adaptive immune response (MAP3K3, IRF8), blood pressure regulation and heart contraction related genes were theorised to be involved with adaptation to extreme temperatures, which may explain the presence of hypoxia (BCL2, CBFA2T3) and heat (HSPH1, MVD) related genes in these cattle. Collectively, these studies show that regions under selection may harbour genes relating to environmental adaption in cattle.

Makina et al. (2015) studied the signatures of selection in six South African Sanga cattle breeds, which includes the Bonsmara and Nguni breeds, using the Illumina BovineSNP50 Bead chip. With the availability of high density SNP data, there is an opportunity to evaluate the genetic structure and selection pressures present in South African cattle, particularly in the Nguni and Bonsmara breeds, as this will serve as a baseline for crossbreeding studies of the reciprocal crosses of these populations. An observation made during hot and dry year (2015/16), when temperatures increased above normal, the Nguni-sired calves performed better than the Bonsmara-sired calves (Pyoos, 2018). Additionally, the identification of genes targeted by selection can be helpful in finding genes related to environmental traits (e.g., genes relating to adaptation) that are difficult to find experimentally. Thus, the aim of this study was to detect signatures of selection in Nguni and Bonsmara cattle using the GGP Bovine 150 K SNP data.

## Materials and Methods

### Ethical Approval

Ethical approval was obtained from the Agricultural Research Council, Animal Production Ethics Committee (APIEC21/06). In addition, approval for the use of the data was obtained from the Bonsmara SA and Nguni Cattle Breeders’ Societies respectively, and the Beef Genomics Programme.

### SNP Data and Quality Control

SNP data generated using the GeneSeek Genomic Profiler 150 K (GGP Bovine 150 K) bead chip of two South African cattle breeds, Nguni (*n* = 258) and Bonsmara (*n* = 248) was used in this study. Individual datasets were merged using PLINK v1.9 (Purcell et al., 2007). Additionally, only autosomal SNPs that were common across all datasets were retained while SNPs present within mitochondrial and sex chromosomes were excluded to avoid any bias. Quality control was performed on the merged data with the parameters involving removal of SNPs with less than 95% call rate; SNPs with less than 2% minor allele frequencies (MAF); samples with more than 10% missing genotypes and SNPs that failed the Hardy Weinberg Equilibrium test (*p* < 0.001). Identity-by-descent (IBD) relatedness between individuals within each population was tested using the “genome” parameter in PLINK v 1.9 (Purcell et al., 2007). For this test, PI_HAT (Proportion of IBD), i.e. P(IBD = 2) + 0.5*P(IBD = 1), where P denotes the probability)—a variable that reveals the extended haplotypes shared between distantly related individuals was determined. Closely related individuals with a PI_HAT value greater than 0.25 were removed.

### Genetic Diversity

The expected heterozygosity (He) and observed heterozygosity (Ho) were computed using the “hardy” parameter in PLINK v1.9 (Purcell et al., 2007) to assess the level of genetic diversity. Briefly, the Ho estimates for each population was determined from observed genotype frequencies obtained as follows: (N–O)/N, where N is the number of non-missing genotypes and O is the number of observed homozygous genotypes for a given individual.

### Population Structure

Principal Component Analysis (PCA) was performed on the Nguni and Bonsmara cattle populations to validate breed separation in the merged (two breeds) datasets. More specifically, the relationship between the first two principal components was examined to show relationship between the animals. The Eigen values were generated using PLINK v1.9, thereafter a PCA plot was generated in R v 4.0.3 (R Core Team, 2019) using the package Tidyverse (Wickham et al., 2019). The estimation of population genetic structure was performed on both populations with the R package LEA (Frichot and François, 2015). Binary files were generated in PLINK v1.9 (Purcell et al., 2007). Thereafter, “.ped” and “.map” files were transformed into the “.geno” format required as input by sNMF using the “ped2geno” function. Individual ancestry coefficients were determined based on the calculation of cross-entropy criterion using the sparse non-negative matrix factorization (snmf) function from the LEA package with K (i.e., number of hypothetical ancestors) values ranging from 1 to 10 with five iterations each. The optimal number of ancestors had the lowest cross-entropy criterion. Finally, the individual ancestry coefficients were plotted using the plot function in R v 4.0.2 (R Core Team, 2019).

### Signatures of Selection

In this study, signatures of selection were identified within and between the two populations. The within population selection signatures was used to show the regions under selection in each of the cattle populations. While the across population selection signatures was used to show the regions under positive selection in both populations. To prepare input files for signatures of selection testing, binary files were created for each breed per chromosome using PLINK v1.9 (Purcell et al., 2007). The pedigree and map files generated were converted to fastPHASE formats using the fcGENE software (Roshyara and Scholz, 2014). The binary files were converted using the “oformat./fastPhase” function in fcGENE, producing the output files in the “.inp” format. Haplotypes for the iHS and XP-EHH analyses were derived with fastPHASE (Scheet and Stephens, 2006) using the output files generated by fcGENE with 10 starts (T10) and 25 iterations (C25) of the expectation-maximization (EM) algorithm. The fastPhase output files were used to detect signatures of selection using the R v 4.0.3 (R Core Team, 2019) and package rehh (Gautier et al., 2017).

The within population signatures of selection in the Nguni and Bonsmara population were determined. The iHS scores were calculated for both the Nguni and Bonsmara using the “ihs2ihs” function in rehh package with the minimum MAF at 0.02. This test detects evidence of recent positive selection at a locus based on the differential levels of LD surrounding a positively selected (derived) allele compared to the background (ancestral) allele at the same position within a population. The iHS candidate regions were identified at *p* < 0.0001, with a window size of 1 Mb allowing for a 10 Kb overlap and the region must contain a minimum of 2 markers. The iHS scores for both the Nguni and Bonsmara were plotted using the “manhattanplot” function from package rehh (Gautier et al., 2017). For visualisation and comparison of selection signals, | iHS| scores were transformed into - log 10 [2Φ - |iHS|] where Φ represents the Gaussian cumulative distribution functionand a value of 4 is equivalent to *p* < 0.0001 (Gautier and Naves, 2011). Cross-population extended haplotype heterozygosity (XP-EHH), statistics was determined to compare EHH profiles between the Nguni and Bonsmara populations. This statistic tests whether the genome site is homozygous in the one population (e.g., Nguni) but polymorphic in the other population (e.g., Bonsmara) through the evaluation of the two populations on one core SNP (Maiorano et al., 2018). The XP-EHH was determined between the Nguni and Bonsmara population using the “ies2xpehh” function in rehh (Sabeti et al., 2007). The candidate regions and plots were identified and visualized with the same parameters as for the iHS test.

Genes contained within the identified candidate genomic regions with signatures of selection have been annotated using the Bovine UMD3.1 reference genome (https://bovinegenome.elsiklab.missouri.edu/). The Bovine QTL database (Hu et al., 2013; http://www.animalgenome.org/cgi-bin/QTLdb/BT/search) was used to identify the overlap with previously published bovine QTLs within the candidate regions. BioMart (Zhang et al., 2011), a program in Ensembl was used to identify genes found within the candidate regions (https://www.ensembl.org/biomart/martview/717be2236eefa78b5aecbc78a6aee7ea).

## Results

### SNP Data and Quality Control

The relatedness test showed no individuals with PI_HAT value greater than 0.25, hence no individuals were removed based on this test. Due to low call rates, 27 Nguni and 32 Bonsmara animals were removed from the dataset. Furthermore, 40,224 and 28,409 SNPs were excluded based on failure to meet the requirements of the missing genotypes, MAF<0.02, the significance of deviation from Hardy Weinberg Equilibrium (*p* < 0.001) in the Nguni and Bonsmara, respectively. Finally, 231 Nguni with 100,952 SNPs and 252 Bonsmara animals with 110,556 SNPs were used for further analysis. A total of 94,147 SNPs were overlapping in the two populations while 6,805 SNPs were unique to the Nguni and 16,419 SNPs were unique to the Bonsmara population.

### Genetic Diversity

The mean He and Ho were higher in the Nguni population compared to the Bonsmara population. The mean He coefficient in the Nguni was 0.363 ± 0.126 while the mean Ho was 0.362 ± 0.130, which was slightly higher compared to the Bonsmara. However, in the Bonsmara population, a difference of 0.003 was observed between the mean He = 0.361 ± 0.132 and the mean Ho = 0.358 ± 0.133.

### Population Structure

**FIGURE 1:**
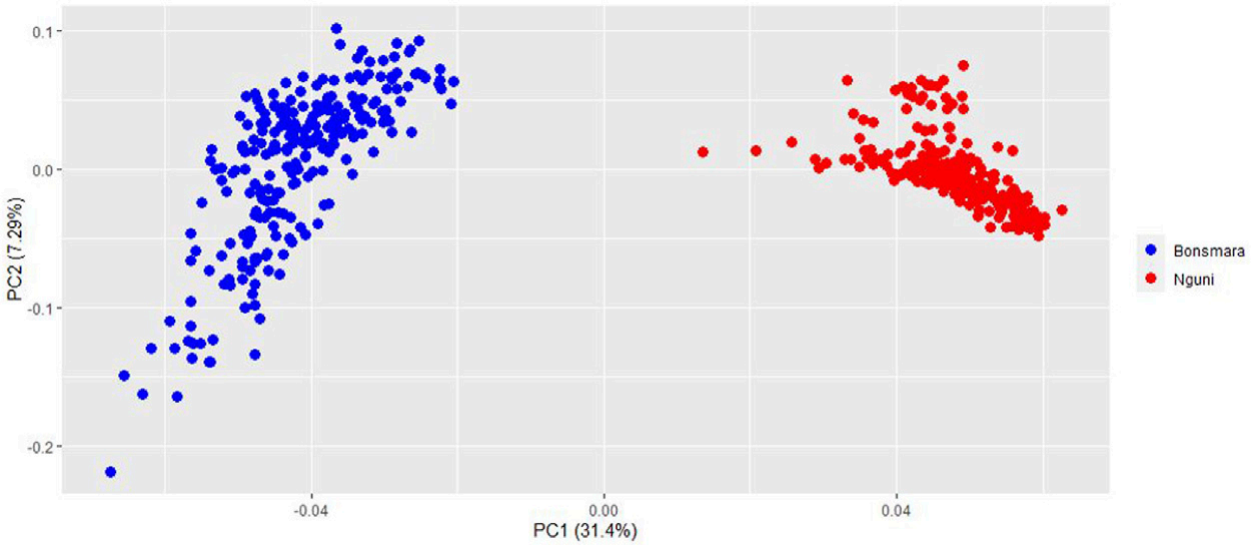
Principal component analysis (PCA) plot showing clustering of Nguni and Bonsmara cattle. Each point represents an individual animal coloured as per breed.

**FIGURE 2:**
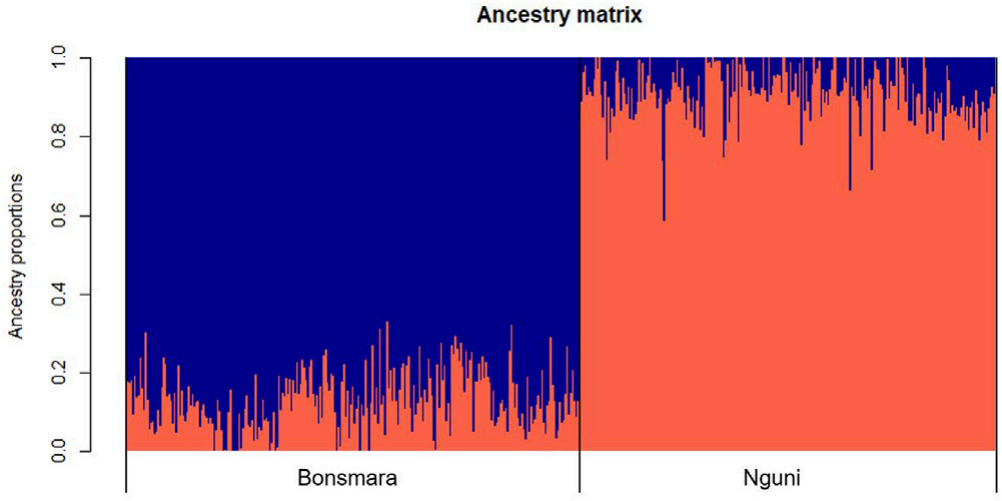
Ancestry matrix of Nguni and Bonsmara populations. Shown in the figure is the ancestry bar plot using sparse nonnegative matrix factorization (sNMF).

The PCA analysis divided the study population into two distinct clusters (Figure 1). The first principal component (PC1) accounted for 31.4% of the total variation and separated the Nguni population from the Bonsmara population. The second principal component (PC2) accounted for 7.59% of the total variation. The clustering was further confirmed by the ancestry matrix of the two populations (Figure 2). For the optimal number of ancestors, the lowest cross-entropy value of 0.851 was chosen with the number of ancestral populations (K) of 2. The ancestry matrix shows that the Nguni population has a different genetic structure when compared to the Bonsmara population.

### Genomic Regions of Signatures of Selection

**FIGURE 3:**
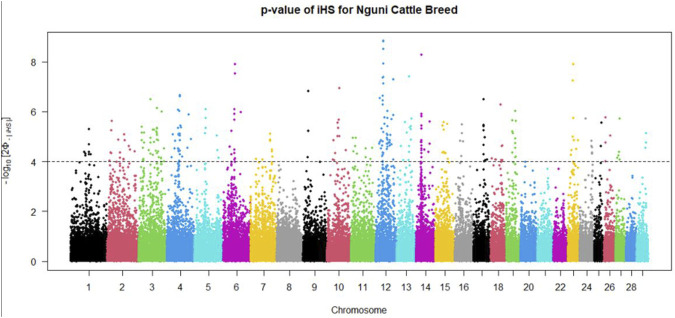
Genome-wide distribution of the integrated haplotype score (iHS) in the Nguni Population. Plot depicting the candidate regions of Nguni population with *p* < 0.0001 indicated with a dotted line The Φ represents the Gaussian cumulative distribution function for iHS Nguni scores.

**FIGURE 4:**
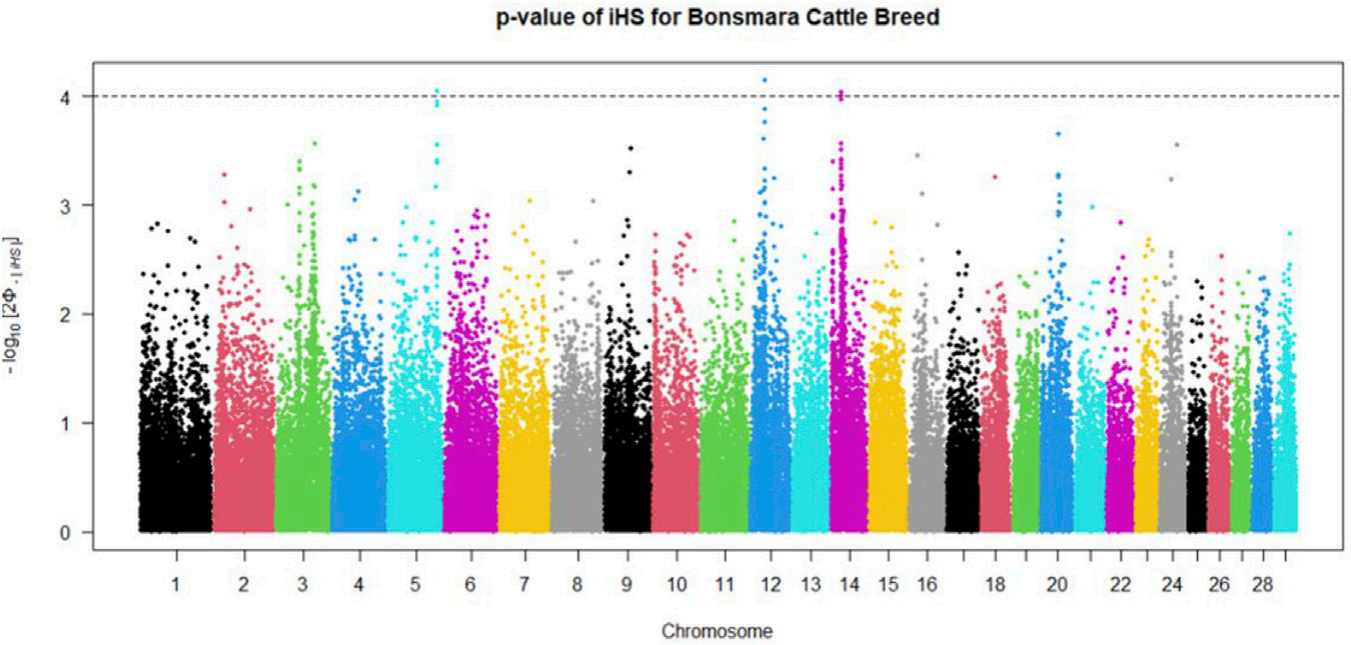
Genome-wide distribution of the integrated haplotype score (iHS) in the Bonsmara Population. Plot depicting the candidate regions of Bonsmara population with *p* < 0.0001 indicated with a dotted line. The Φ represents the Gaussian cumulative distribution function for iHS Bonsmara scores.

**FIGURE 5:**
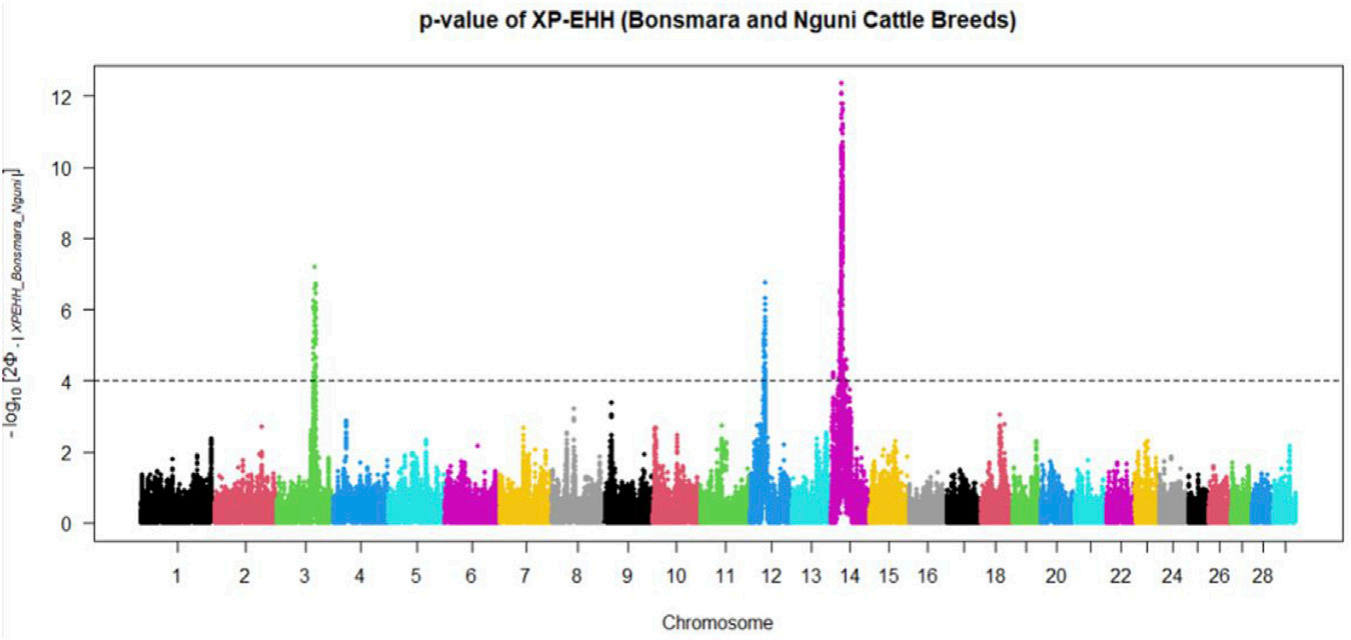
Manhattan plots showing the candidate regions of cross-population extended haplotype homozygosity (XP-EHH) analysis between Bonsmara and Nguni cattle populations with the *p* < 0.0001 threshold indicated with a dotted line. The Φ represents the Gaussian cumulative distribution function for XP-EHH scores.

Positive signatures of selection were identified within and across the Nguni and Bonsmara populations. The iHS analysis for the Nguni revealed 118 regions of selection across all chromosomes except on BTA 8, 20, 21, 22, and 28 (Figure 3). The highest iHS score of 6.047 was located on BTA 12 (*p* < 0.0001). In contrast to the Nguni, only three significant regions were found for the Bonsmara. The iHS analysis for Bonsmara only identified regions on BTA 5, 12 and 14 (*p* < 0.0001, Figure 4). For the across population analysis, three regions of selection were identified across the two populations located on BTA 3, 12 and 14 (*p* < 0.0001, Figure 5). The highest XP-EHH score of 7.243 was detected on BTA14. The genes identified within the candidate regions are summarised in Table 1 and Supplementary Table S1. Additionally, all the putative genes identified across all candidate are summarized in Supplementary Table S2.

**TABLE 1:** Genomic candidate regions detected by integrated haplotype score (iHS) and cross-population extended haplotype homozygosity (XP-EHH) analysis in the Nguni and Bonsmara population, *p* < 0.0001.

Biological role	BTA	Gene start^a^	Gene end^a^	Gene Name	Reference
iHS nguni					
Heat Tolerance	15	53393791	53406519	DNAJB13	Linde (2018)
	19	42257571	42258084	HSPB9	Makina et al. (2015)
	23	17752495	17758156	HSP90AB1	Charoensook et al. (2012)
DNA Methylation	2	95910212	95920426	METTL21A	Jakobsson et al. (2016)
	5	106319032	106389988	PRMT8	Do et al. (2017)
	12	78920744	78929824	METTL21C	Huang et al. (2014)
	13	62142537	62176363	DNMT3B	Guo et al. (2012)
	17	44257597	44259224	MBD3L1	Jin et al. (2008)
Feed efficiency	15	54737997	54748417	SERPINH1	Sun et al. (2019)
	18	51865091	51878472	PLAUR	Sun et al. (2019)
	18	52695747	52724172	RELB	Sun et al. (2019)
Nitrogen Metabolism	5	101041433	101090609	A2ML1	Wang et al. (2021)
	3	23536511	23560620	HMGCS2	Wang et al. (2021)
	18	52637233	52640572	APOE	Wang et al. (2021)
	19	41834618	41840426	KRT17	Wang et al. (2021)
XP-EHH Nguni and Bonsmara					
Heat Tolerance	14	30355171	30448182	DNAJC5B	Bhuiyan et al. (2018)
	12	29796159	29819628	HSPH1	Howard et al. (2014)
DNA Methylation	12	35796991	35817783	EEF1AKMT1	Hamey and Wilkins (2018)
Feed Efficiency	14	24365744	24432635	FAM110B	Singh et al. (2020)
	14	24587138	24624435	UBXN2B	Singh et al. (2020)

a Gene start and end position are reported in base pairs (bp).

Overlapping regions were further interrogated through the comparison of all the regions of selection identified in this study. Based on the criteria of Voight et al. (2006), the overlapping regions of selection are defined as those regions located above the cut-off threshold and in the same chromosomal location. Two significant selection regions were detected by iHS Bonsmara, iHS Nguni and XP-EHH tests. The two regions were detected on BTA12 (32.2–34.1 Mb) and BTA14 (23.5–25.4 Mb) (Table 2). Furthermore, one overlapping region was detected by the iHS analysis between the Nguni and Bonsmara (Table 3).

**TABLE 2:** Genes and QTLs found in the overlapping regions detected by integrated haplotype score (iHS) and cross-population extended haplotype homozygosity (XP-EHH) in the analysis Nguni and Bonsmara cattle (*p* < 0.0001).

BTA	Start (Mb)	End (Mb)	No. genes	Gene	QTLs relating to			
					Meat quality	Diseases	Performance	Reproduction
12	32.2	34.1	19	GSX1, POLR1D, LNX2, MTIF3, GTF3A, RASL11A, RPL21, USP12, GPR12, WASF3, CDK8, RNF6, SHISA2, 5S_rRNA, ATP8A2, U6, NUP58, MTMR6, AMER2	Meat color L*(QTL:19,847), Lean meat yield (QTL:37,145)	Clinical mastitis (QTL:157,300), Bovine *tuberculosis* susceptibility (QTL:167,804)	Carcass weight (QTL:24,628), Feed conversion ratio (QTL:11,456), Average daily feed intake (QTL:21,005)	
14	23.5	25.4	11	SDR16C6, PENK, U6, BPNT2, FAM110B, UBXN2B, CYP7A1, U1, SDCBP, NSMAF, TOX	Intramuscular fat (QTL:122,426), Longissimus muscle area (QTL:215,146), Shear force (QTL:106,395), Subcutaneous rump fat thickness (QTL:15,255)	Stillbirth (QTL:15,030), Hoof and leg disorders (QTL:122,502)	Dry matter intake (QTL:213,147), Feed conversion ratio (QTL:213,190), Growth index (QTL:102,033), Marbling score (QTL:122,425), Residual feed intake (QTL:20,842), Residual gain (QTL:213,210)	Scrotal circumference (QTL:30,637), Sexual precocity (QTL:169,861), Stature (QTL:16,284)

**TABLE 3:** One overlapping region detected by comparing integrated haplotype score (iHS) analysis in Nguni and iHS analysis Bonsmara populations (*p* < 0.0001).

BTA	Start (Mb)	End (Mb)	Genes common to both	Genes unique to Nguni	QTLS relating to		
					Growth	Reproduction	Disease
5	105.6	107.5	PARP11, CRACR2A, PRMT8, TSPAN11, TSPAN9, TEAD4, RHNO1, FOXM1, TEX52, NRIP2, ITFG2, FKBP4, WASHC1, IQSEC3	C5H12orf4, FGF6, FGF23, TIGAR, CCND2, SLC6A12, SLC6A13, KDM5A, CCDC77, B4GALNT3, SNORA70, NINJ2	Average daily gain (QTL:164,654)	Stature (QTL:154,109)	Bovine respiratory disease susceptibility (QTL:160,084)
					Metabolic body weight (QTL:188,728)	Pelvic area (QTL:106,466)	Dystocia (QTL:14,701)
					Length of productive life (QTL:122,677)	Scrotal circumference (QTL:139,011)	Bovine *tuberculosis* susceptibility (QTL:167,792)
					Body weight gain (QTL:67,109)	Calf size (QTL:30,494)	
					Hip height (QTL:172,045)	Calving ease (QTL:40,773)	
						Conception rate (QTL:176,997)	
						Daughter pregnancy rate (QTL:40,769)	
						Lactation persistency (QTL:125,220)	

## Discussion

Genetic exploration of beef cattle breeds can provide vital information that may contribute to sustainable meat production systems such as crossbreeding. The main goal was to unravel the genetic variability and determine the signatures of selection in Nguni, an indigenous cattle breed and the Bonsmara, a composite breed using bovine 150 K SNP data. The results of this study showed that both Nguni and Bonsmara populations have a high level of genetic diversity. More specifically, the mean expected heterozygosity values were comparable in Nguni (He = 0.362) and Bonsmara (He = 0.361) populations (Table 1). In addition, the level of genetic diversity in Bonsmara populations was similar to those reported by Bosman et al. (2017) with He = 0.365. However, the Nguni population had higher level of genetic diversity than previously reported by Makina et al. (2014) with He = 0.28, although this may be due to the low number of samples in the previous study. Furthermore, PCA and ancestry matrix analyses agreed with previous findings of Makina et al. (2016), where the Nguni cluster is distinct from the Bonsmara cluster (Figures 1, 2). These PCA results are also similar to those found by Madilindi et al. (2020), where the South African Nguni population clustered away from the Bonsmara reference population when using microsatellite data. Overall, these findings display that the Nguni population is significantly different to the Bonsmara, and each population is genetically variable.

Candidate regions that show preferential selection in the genome of Nguni and Bonsmara cattle were discovered using the two statistical methods (iHS and XP-EHH), successfully applied in multiple studies to identify signatures of selection in cattle (Bahbahani et al., 2018; Cheruiyot et al., 2018; Alshawi et al., 2019; Aliloo et al., 2020). While the iHS analysis detected 118 regions of selection within the Nguni population, only three regions of selection signatures were found within the Bonsmara population. This may be due to Nguni experiencing selection pressures over longer period of time than the Bonsmara cattle (Makina et al., 2015). The XP-EHH analysis across the Nguni and Bonsmara population identified 3 regions under selection (BTA 3, 12 and 14) that were previously found to be under selection. For example, the study by Ben-Jemaa et al. (2020) detected the same region (BTA 12:32.2-34.1 Mb) using the XP-EHH tests in Indicine (Gir and Nelore) vs. North African cattle analysis. Another study by Bahbahani et al. (2015) identified the same region on BTA 12: 32.2-34.1 Mb comparing East African Shorthorn Zebu (EASZ) vs. European Taurine (Holstein-Friesian and Jersey) and comparing EASZ vs. African Taurine (N’Dama). Cheruiyot et al. (2018) also identified the region on BTA 14: 23.49-23.86 Mb through XP-EHH analysis in Tanzanian crossbred cattle vs. EASZ. The genes detected in this region include, PLAG1, CHCHD&, TOX, and TMEM68 involved in pleiotropic traits associated with milk and growth in cattle. Reported studies displayed regions of signatures of selection present on BTA 12 and 14 and coincide with the findings of this study.

Climate change may cause increased thermal stress experienced by livestock species, resulting in reduced meat production and decreased fertility that will have a negative impact on food security (Sejian et al., 2018). In this study, several genes related to heat stress were identified in the genomic regions displaying positive signatures of selection in both populations. This is important, as heat stress is the physiological imbalance between the heat produced within the body and its expenditure, overall negatively impacting growth, milk production and reproduction (Belhadj Slimen et al., 2016). Genes relating to heat shock included HSP90 which is one of the members of the heat shock protein family that plays a key role in influencing heat stress in animals. Charoensook et al. (2012) identified polymorphisms in the bovine HSP90AB1 associated with heat tolerance in Thai indigenous cattle, which may support the presence of *HSP90AB1* (BTA23: 17.75-17.75 Mb) in the Nguni cattle, and not being detected in the Bonsmara cattle. Another *DnaJ heat shock protein family (Hsp40) member B13* (DNAJB13) gene located on BTA 15: 53.39-53.40 Mb was found in the Nguni population. However, this gene was shown to be expressed in both Nguni and Bonsmara populations during low energy diet treatment (Linde, 2018). The *heat shock protein family H (Hsp110) member 1* (HSPH1) detected on BTA12: 29.79- 29.81 Mb has shown to be involved in Nguni and Bonsmara body temperature regulation in the presence of climatic stress (Howard et al., 2014). *Heat shock protein family B (small) member 9* (HSPB9) located on BTA 19 was previously detected in Nguni cattle and showed to be associated to tropical adaptation in Zebu cattle (Makina et al., 2015). This region (BTA 19: 42.25-42.26 Mb) is under selection in the current Nguni population. The expression of additional heat shock proteins in the Nguni could play a role in its ability to tolerate high temperatures better than the Bonsmara cattle.

DNA methylation is one of the necessary processes involved in epigenetic gene regulation (Tian et al., 2017). Hence, genes relating to DNA methylation were also located in the regions displaying signatures of selection. The *de novo* and maintenance methylation reactions carried out by DNA methyl transferases have been studied extensively (Chen and Li, 2006; Yakovlev, 2018). Despite the stable and heritable features of DNA methylation patterns, genome-wide DNA demethylation occurs both in developing germ cells and in fertilized oocytes. Mbd3l1 expressed predominantly in testis and found specifically in round spermatids was identified in Nguni cattle (BTA17: 44.25-44.26 Mb). Expression of Mbd3l1 is specific to haploid male germ cells. This protein could be incorporated into sperm chromatin, and after fertilization could participate in demethylation of the paternal genome (Jin et al., 2008). *Methyltransferase like 21A* (METTL21A) located on BTA2, is responsible for the methylation of HSPA1-K561, a heat shock protein that was identified in the Nguni population. This interaction displays the vital role of DNA methylation in response to heat stress (Jakobsson et al., 2016). *Protein arginine methyl transferase 8* (PRMT8) was shown to play a role in lactation persistency in Canadian Holstein cattle and was found in both Nguni and Bonsmara cattle, located on BTA 5 (Do et al., 2017). This further supports the detection of Lactation persistency (QTL: 125,220) located on BTA 5:105.6-107.5 Mb. *Methyltransferase like 21C* (METTL21C), located on BTA12 was shown to be differently expressed in Bonsmara and Nguni breeds by Linde (2018). However, in this study, METTL21C was detected only in the Nguni cattle. Additionally, METTL21C showed to be an important modulator of protein degradation in skeletal muscle in mice by Wiederstein et al. (2018). The XP-EHH revealed BTA12: 35.79-35.81 Mb to harbour EEF1AKMT1 to play a role in DNA methylation in both populations. The presence of DNA methylation regulating genes in the Nguni and Bonsmara are important for its adaptation to thermally challenged environments.

Maternal effects, which result from variation in the environment provided by dams to their progeny, are recognized as being important for a variety of traits expressed at, and after birth in beef cattle (Campêlo et al., 2004; Zhang et al., 2021). In addition, the study by Linde (2018) showed that the impact of a low energy diet on the expression of *DNA methyltransferase 3B* (DNMT3B) increased expression in the Bonsmara compared to the Nguni. However, in this study, DNMT3B located on BTA 13: 62.14-62.17 Mb was only identified in the Nguni population. This could possibly imply that the DNMT3B is under strong selection in the Nguni population and not in the Bonsmara. An interesting finding by Pyoos (2018) showed the maternal effect of the Nguni on birth weight and pre-weaning growth was lower compared to the Bonsmara, possibly due to reduced feed intake on the part of the Nguni cow and this could be another reason for the detection of DNMT3B only in the Nguni population.

Animal growth is dependent on feed intake and metabolism of feed into useful energy for the animal (Dong et al., 2019). Furthermore, the need for animals to utilize feed efficiently is a necessity in order to reduce secondary by-products such as methane emissions that are harmful to the environment (Dong et al., 2019). In this study candidate regions that display signatures of selection, include QTLs associated with residual feed intake, conception rate, body weight gain, carcass weight, residual feed intake and heat tolerance. The two overlapping regions that were detected by all EHH analysis located on BTA 12 and 14, include QTLs associated with traits such as immunity, meat quality, disease, growth, and reproduction. For instance, the QTL for feed conversion ratio (FCR) (QTL: 213,190) located on BTA14 was common across both populations, implying that both breeds have the ability to convert fibrous diet into reasonable weight gain (Shike, 2013). Jiyana (2019) found that the Nguni need less feed than the Bonsmara to gain the same weight. This is in line with the ability of the Nguni breed to survive on pasture grazing for a long time while maintaining its weight (Nyamushamba et al., 2017). The impact of using Nguni cattle with lower FCR in crossbreeding programs may reduce the amount of feed needed to sustain them for longer periods. This observation is supported by the feed efficiency genes such as *serpin family H member 1* (SERPINH1, BTA15: 54.73-54.74 Mb), *plasminogen activator, urokinase receptor* (PLAUR, BTA18:51.86-51.87 Mb), *RELB proto-oncogene, NF-kB subunit* (RELB, BTA18:52.69-52.72 Mb) detected to be under selection in the Nguni population which have previously been detected. SERPINH1, PLAUR and RELB have shown to be associated to feed conversion ratio, dry matter intake, average daily gain and residual feed intake in beef cattle (Sun et al., 2019). Additionally, the XP-EHH analysis revealed two genes, the *family with sequence similarity 110 member B* (FAM110B) and *UBX domain protein 2B* (UBXN2B) relating to feed efficiency to be found on BTA 14:24.36-24.62 Mb in both Nguni and Bonsmara. For sufficient feed utilization, nitrogen metabolism plays a key role in the animal’s utilization of nitrogen from feed. In this study several genes under selection relating to nitrogen metabolism were identified only in the Nguni population. *Keratin 17*(KRT17) located on BTA19:41.83-41.84 Mb plays a role in the regulation of urine urea nitrogen yield and urine volume, while during milk protein synthesis *Apolipoprotein E* (APOE) located on BTA18:52.63-52.64 Mb improved ribosome biosynthesis resulting in less nitrogen being excreted as urine urea as previously detected by Wang et al. (2021). Other genes include *Alpha-2-macroglobulin like 1* (A2ML1 located on BTA5:101.04-101.09 Mb) and *3-hydroxy-3-methylglutaryl-CoA synthase 2* (HMGCS2 located on BTA3: 23.53-23.56 Mb) were found to increase urine urea nitrogen and also previously detected by Wang et al. (2021). Previous studies observed that Nguni cattle were more capable of maintaining their body weight during winter than other breeds. Moreover they had higher blood urea (N) and ruminal ammonia levels (Linington, 1990) than other breeds. Ultimately, based on the presence of these genes in the Nguni and Bonsmara cattle, it clearly shows that they may be involved with effective feed regulation while also reducing the impact of nitrogen released into the environment.

## Conclusion

With the use of high-density genome-wide SNP genotype data it was possible to understand the genetic diversity, population structure and selection signatures present in the South African Nguni and Bonsmara cattle populations. Broadly, the findings from this study revealed that these two populations are genetically distinct and have different selection signatures influencing their population structure. Candidate regions under positive selection were associated with heat tolerance, feed efficiency, nitrogen metabolism and DNA methylation. The QTLs relating to meat quality, disease resistance, growth, reproduction and immunity were located in the candidate regions under selection in the Nguni and Bonsmara populations. Furthermore, the results also indicate that different ancestral backgrounds (indigenous *vs*. composite breed genotypes) are advantageous in different regions of the genome, thus, they may assist in designing breeding programs to enhance performance of both the Nguni and Bonsmara cattle, as well as using the two breeds in crossbreeding programs. The genes identified may be used in improving the current Nguni and Bonsmara cattle population through controlled breeding strategies and selection of specific traits. The findings in this study present an opportunity in identifying causative variants that confer adaptation of indigenous and composite cattle populations to the South African environment.

## Data Availability

The datasets presented in this article are not readily available because of a binding memorandum of agreement between the South African Stud Book (SA Studbook) and the Agricultural Research Council (ARC). Requests to access the datasets should be directed to SA Studbook.
