# Supplementary material for: Selection Signatures in South African Nguni and Bonsmara Cattle Populations Reveal Genes Relating to Environmental Adaptation
Source: Front Genet. 2022 Jun 17;13:909012. doi: 10.3389/fgene.2022.909012 (PMC9247466; doi:10.3389/fgene.2022.909012)
Supplement: Supplementary file 1 [file DataSheet1.docx]

Supplementary Material

**Supplementary Table 1**: Other candidate regions detected by integrated haplotype score (iHS) and cross-population extended haplotype homozygosity (XP-EHH) analysis in the Nguni and Bonsmara population, P<0.0001.

| **BTA** | **Gene start (bp)** | **Gene end (bp)** | **Gene name** | **Reference** | **Biological Role** |
| --- | --- | --- | --- | --- | --- |
| **iHS Nguni** | | | |  |  |
| 1 | 80643739 | 80661936 | DNAJB11 | (Ajayi *et al.,* 2018) | Heat Tolerance |
| 3 | 66664210 | 66707306 | DNAJB4 | (Ajayi *et al.,* 2018) |  |
| 4 | 59583232 | 59586115 | DNAJB9 | (Ajayi *et al.,* 2018) |  |
| 7 | 49894161 | 49909804 | HSPA9 | (Ajayi *et al.,* 2018) |  |
| 7 | 50680729 | 50712129 | DNAJC18 | (Ajayi *et al.,* 2018) |  |
| 10 | 36316703 | 36347349 | DNAJC17 | (Ajayi *et al.,* 2018) |  |
| 12 | 29796159 | 29819628 | HSPH1 | (Tripathy *et al.,* 2021;  Zhang *et al.,* 2011b) |  |
| 13 | 29491860 | 29517411 | HSPA14 | (Tripathy *et al.,* 2021;  Zhang *et al.,* 2011b) |  |
| 13 | 53907615 | 53936817 | DNAJC5 | (Ajayi *et al.,* 2018) |  |
| 13 | 66086809 | 66143601 | MROH8 | (Edea *et al.,* 2020) |  |
| 18 | 46466912 | 46469531 | HSPB6 | (Kumar *et al.,* 2015a) |  |
| 19 | 42139364 | 42169593 | DNAJC7 | (Ajayi *et al.,* 2018) |  |
| 23 | 27520317 | 27522790 | HSPA1A | (Pires *et al.,* 2019; Kumar *et al.,* 2015b; Chulayo *et al.,* 2016) |  |
| 23 | 27523225 | 27527209 | HSPA1L | (Ortega *et al.,* 2016; Stricher *et al.,* 2013) |  |
| 25 | 33573623 | 33575091 | DNAJC30 | (Vasudhar *et al.,* 2020) |  |
| 3 | 14098949 | 14105919 | RRNAD1 | (Boitard *et al.,* 2021) | DNA Methylation |
| 3 | 15121790 | 15298435 | ASH1L | (Doherty *et al.,* 2014) |  |
| 3 | 19801955 | 19834258 | SETDB1 | (Golding *et al.,* 2015) |  |
| 5 | 107225327 | 107290351 | KDM5A | (Pegolo *et al.,* 2017) |  |
| 7 | 49749644 | 49805938 | KDM3B | (Dolebo *et al.,* 2019) |  |
| 9 | 25253397 | 25317010 | TRMT11 | (Lim *et al.,* 2013) |  |
| 10 | 26119686 | 26125451 | METTL17 | (Shi *et al.,* 2019) |  |
| 16 | 37440402 | 37442731 | METTL18 | (Nemcova *et al.,* 2016) |  |
| 17 | 53650953 | 53768578 | KDM2B | (Liu *et al.,* 2018) |  |
| 23 | 27467178 | 27480388 | EHMT2 | (Li *et al.,* 2020) |  |
| 23 | 39389854 | 39432004 | KDM1B | (Cui *et al.,* 2018) |  |
| 24 | 53580798 | 53648542 | MBD2 | (Caballero *et al.,* 2009) |  |
| 14 | 24365744 | 24432635 | FAM110B | (Singh *et al*., 2020) | Feed Efficiency |
| 14 | 24587138 | 24624435 | UBXN2B | (Singh *et al*., 2020) |  |
| 4 | 27093041 | 27667961 | HDAC9 | (Olivieri *et al*., 2016) |  |
| 11 | 9209287 | 9210408 | GPR45 | (Olivieri *et al*., 2016) |  |
| 11 | 9215779 | 9243006 | TGFBRAP1 | (Olivieri *et al*., 2016) |  |
| 11 | 9268665 | 9316088 | FHL2 | (Olivieri *et al*., 2016) |  |
| 11 | 9706676 | 9718212 | POLE4 | (Olivieri *et al*., 2016) |  |
| 11 | 9743172 | 9810098 | HK2 | (Olivieri *et al*., 2016) |  |
| 23 | 17801845 | 17813188 | AARS2 | (Olivieri *et al*., 2016) |  |
| 7 | 24410759 | 24678664 | ADAMTS19 | (Seabuy *et al*., 2017) |  |
| 13 | 54054302 | 54055810 | TNFRSF6B | (Sun *et al*., 2019) |  |
| 13 | 62565844 | 62575200 | BPIFA2C | (Wang *et al*., 2021) | Nitrogen Metabolism |

**Supplementary Table 2:** All putative genes found in the candidate genes detected by integrated haplotype score (iHS) and cross-population extended haplotype homozygosity (XP-EHH) analysis in the Bonsmara and Nguni Populations at P<0.0001.

| **Test** | **BTA** | **Start** | **End** | **Length (Mb)*** | **Genes** |
| --- | --- | --- | --- | --- | --- |
| iHS Bonsmara  P<0.0001 | 5 | 106000000 | 108000000 | 2 | PARP11, CRACR2A, PRMT8, TSPAN11, TSPAN9, TEAD4, RHNO1, FOXM1, TEX52, NRIP2, ITFG2, FKBP4, WASHC1, IQSEC3 |
|  | 12 | 32200000 | 34100000 | 1.9 | GSX1, POLR1D, LNX2, MTIF3, GTF3A, RASL11A, RPL21, USP12, GPR12, WASF3, CDK8, RNF6, SHISA2, 5S_rRNA, ATP8A2, U6, NUP58, MTMR6, AMER2 |
|  | 14 | 23500000 | 25400000 | 1.9 | SDR16C6, PENK, U6, BPNT2, FAM110B, UBXN2B, CYP7A1, U1, SDCBP, NSMAF, TOX |
| XP-EHH Bonsmara vs Nguni  P<0.0001 | 3 | 80400000 | 86300000 | 5.9 | JAK1, RAVER2, CACHD1, UBE2U, ROR1, U5, PGM1, EFCAB7, ITGB3BP, 5S_rRNA, ALG6, FOXD3, 7SK, ATG4C, DOCK7, ANGPTL3, USP1, KANK4, U6, PATJ, TM2D1, NFIA, U1, C3H1orf87, HOOK1, FGGY |
|  | 12 | 29300000 | 31800000 | 2.5 | bta-mir-2299, B3GLCT, HSPH1, TEX26, MEDAG, ALOX5AP, USPL1, HMGB1, KATNAL1, SNORA70, UBL3, SLC7A1, MTUS2, POMP, FLT1, bta-mir-2300a |
|  | 12 | 32100000 | 3.6e+07 | 3.9 | PDX1, GSX1, POLR1D, LNX2, MTIF3, GTF3A, RASL11A, RPL21, USP12, GPR12, WASF3, CDK8, RNF6, SHISA2, 5S_rRNA, ATP8A2, U6, NUP58, MTMR6, AMER2, C1QTNF9, MIPEP, TNFRSF19, SACS, SGCG, FGF9, MICU2, ZDHHC20, MRPL57, SKA3, SAP18, LATS2, XPO4, EEF1AKMT1, IL17D, IFT88, CRYL1 |
|  | 14 | 5400000 | 7600000 | 2.2 | KHDRBS3, bta-mir-30d, bta-mir-30b, ZFAT |
| XP-EHH Bonsmara vs Nguni  P<0.0001 | 14 | 18600000 | 20500000 | 1.9 | CEBPD, SPIDR, H3-5, PRKDC, MCM4, UBE2V2, EFCAB1, SNAI2, PPDPFL, SNTG1 |
|  | 14 | 22100000 | 30600000 | 8.5 | SOX17, RP1, XKR4, TMEM68, TGS1, LYN, RPS20, MOS, PLAG1, CHCHD7, SDR16C5, SDR16C6, PENK, BPNT2, FAM110B, UBXN2B, CYP7A1, U1, SDCBP, NSMAF, TOX, CA8, RAB2A, CHD7, U6, CLVS1, ASPH, 5S_rRNA, NKAIN3, SNORA70, GGH, TTPA, YTHDF3, bta-mir-124a-2, CYP7B1 |
|  | 14 | 32800000 | 34700000 | 1.9 | SULF1, SLCO5A1, , PRDM14, NCOA2, bta-mir-2285bw, U6, TRAM1, LACTB2, XKR9 |
| iHS Nguni  P<0.0001 | 1 | 60000000 | 61900000 | 1.9 | GAP43, LSAMP, 7SK |
|  | 1 | 63700000 | 66700000 | 3 | IGSF11, UPK1B, B4GALT4, ARHGAP31, TMEM39A, POGLUT1, TIMMDC1, CD80, ADPRH, POPDC2, COX17, CFAP91, NR1I2, GSK3B, GPR156, LRRC58, FSTL1, NDUFB4, HGD, RABL3, GTF2E1, SNORA70, STXBP5L, POLQ, FBXO40, HCLS1, GOLGB1, IQCB1, EAF2, ILDR1, CD86, CASR |
|  | 1 | 79200000 | 82300000 | 3.1 | BCL6, RTP2, SST, RTP4, MASP1, RTP1, ST6GAL1, bta-mir-2285k-3, ADIPOQ, RFC4, EIF4A2, SNORA63, bta-mir-1248-1, KNG1, HRG, FETUB, AHSG, DNAJB11, TBCCD1, CRYGS, DGKG, ETV5, TRA2B, IGF2BP2, SENP2, LIPH, 5S_rRNA, TMEM41A, MAP3K13, EHHADH, C1H3orf70, VPS8, U6 |
|  | 1 | 87200000 | 89100000 | 1.9 | PEX5L, USP13, NDUFB5, MRPL47, ACTL6A, GNB4, MFN1, ZNF639, KCNMB3, PIK3CA, ZMAT3, KCNMB2, PLCXD1, GTPBP6 |
|  | 2 | 10700000 | 12600000 | 1.9 | FSIP2, U1, ZNF804A |
|  | 2 | 16100000 | 19100000 | 3 | CWC22, ZNF385B, SESTD1, CCDC141, PLEKHA3, FKBP7, PJVK, PRKRA, OSBPL6, RBM45, CYCT, PDE11A |
|  | 2 | 20500000 | 22400000 | 1.9 | MTX2, HOXD1, HOXD3, HOXD4, bta-mir-10b, HOXD8, HOXD9, HOXD10, HOXD11, HOXD12, HOXD13, EVX2, LNPK, ATP5MC3, ATF2, CHN1, CHRNA1, WIPF1, GPR155, SCRN3, CIR1 |
|  | 2 | 49000000 | 50900000 | 1.9 |  |
|  | 2 | 55500000 | 57500000 | 2 | LRP1B |
|  | 2 | 72200000 | 75200000 | 3 | GLI2, TFCP2L1, CLASP1, NIFK, TSN |
|  | 2 | 76100000 | 78000000 | 1.9 | CNTNAP5 |
|  | 2 | 94900000 | 96800000 | 1.9 | ADAM23, FAM237A, DYTN, MDH1B, FASTKD2, CPO, KLF7, CREB1, METTL21A, 5S_rRNA, CCNYL1, FZD5, PLEKHM3, CRYGD, CRYGC, CRYGB, CRYGA, C2H2orf80, bta-mir-2285ax-2, IDH1, PIKFYVE, PTH2R |
|  | 2 | 99000000 | 100900000 | 1.9 | ERBB4, 7SK |
|  | 2 | 119800000 | 121700000 | 1.9 | DIS3L2, ALPI, ECEL1, PRSS56, CHRND, CHRNG, EIF4E2, PHC2, ZNF362, TRIM62, AZIN2, AK2, bta-mir-2357, RNF19B, TMEM54, HPCA, FNDC5, S100PBP, YARS1, KIAA1522, SYNC, RBBP4, ZBTB8OS, ZBTB8A, BSDC1, TSSK3, FAM229A, MARCKSL1, HDAC1, LCK, FAM167B, MTMR9, EIF3I, TMEM234, DCDC2B, IQCC, CCDC28B, U6, CLDN12, SHOX, bta-mir-2887-1 |
| iHS Nguni  P<0.0001 | 3 | 13000000 | 15900000 | 2.9 | ETV3, ETV3L, ARHGEF11, LRRC71, PEAR1, NTRK1, INSRR, SH2D2A, PRCC, HDGF, MRPL24, RRNAD1, ISG20L2, CRABP2, NES, BCAN, HAPLN2, GPATCH4, bta-mir-6534, NAXE, TTC24, IQGAP3, MEF2D, RHBG, TSACC, CCT3, GLMP, TMEM79, SMG5, PAQR6, BGLAP, PMF1, SLC25A44, SEMA4A, LMNA, MEX3A, RAB25, LAMTOR2, UBQLN4, SSR2, ARHGEF2, KHDC4, RIT1, SYT11, GON4L, MSTO1, DAP3, ASH1L, RUSC1, FDPS, PKLR, HCN3, CLK2, SCAMP3, FAM189B, GBA, MTX1, THBS3, MUC1, TRIM46, KRTCAP2, DPM3, SLC50A1, EFNA1, EFNA3, EFNA4, ADAM15, DCST1, ZBTB7B, FLAD1, CKS1B, SHC1, PBXIP1, PMVK, U6, KCNN3 |
|  | 3 | 18600000 | 24800000 | 6.2 | TCHHL1, bta-mir-2284z-4, 7SK, S100A11, S100A10, bta-mir-2412, THEM4, THEM5, C2CD4D, RORC, LINGO4, TDRKH, OAZ3, MRPL9, CELF3, SNX27, TUFT1, CGN, POGZ, PSMB4, SELENBP1, RFX5, PI4KB, ZNF687, U6, PSMD4, PIP5K1A, VPS72, TMOD4, SCNM1, LYSMD1, TNFAIP8L2, SEMA6C, GABPB2, MLLT11, CDC42SE1, C3H1orf56, BNIPL, PRUNE1, MINDY1, ANXA9, CERS2, SETDB1, ARNT, CTSK, CTSS, HORMAD1, GOLPH3L, ENSA, MCL1, ADAMTSL4, ECM1, TARS2, RPRD2, PRPF3, bta-mir-12064, MRPS21, CIART, C3H1orf54, APH1A, CA14, ANP32E, PLEKHO1, VPS45, OTUD7B, MTMR11, SF3B4, SV2A, BOLA1, H2AC20, H2AC21, H2AC19, H4C14, H2AC18, H3C13, H2BC18, FCGR1A, MGC134040, U1, HJV, TXNIP, POLR3GL, ANKRD34A, RBM8A, PEX11B, ITGA10, ANKRD35, PIAS3, NUDT17, POLR3C, RNF115, PDZK1, GPR89A, GJA8, GJA5, ACP6, 7SK, BCL9, OR13L2, OR13L12, U6, CHD1L, FMO5, PRKAB2, PDE4DIP, SEC22B, NOTCH2, REG4, HMGCS2, PHGDH, ZNF697, HSD3B1, HAO2, 7SK, WARS2, TBX15 |
|  | 3 | 45500000 | 47400000 | 1.9 | DPYD, PTBP2, U3, U6 |
|  | 3 | 51300000 | 53600000 | 2.3 | BTBD8, EPHX4, BRDT, TGFBR3, CDC7, HFM1, ZNF644, bta-mir-2285b-2, BARHL2, ZNF326, LRRC8D, bta-mir-2285k-5, LRRC8C, |
|  | 3 | 63000000 | 64900000 | 1.9 | ADGRL2, U6 |
|  | 3 | 66400000 | 68400000 | 2 | GIPC2, U6, DNAJB4, U6, FUBP1, NEXN, MIGA1, USP33, ZZZ3, AK5, PIGK, ST6GALNAC5 |
|  | 3 | 74400000 | 76300000 | 1.9 | CTH, ANKRD13C, SRSF11, LRRC40, LRRC7 |
|  | 3 | 78500000 | 82600000 | 4.1 | DNAI4, INSL5, DYNLT5, SGIP1, PDE4B, MGC137454, LEPR, LEPROT, DNAJC6, AK4, bta-mir-101-1, JAK1, RAVER2, CACHD1, UBE2U, ROR1, U5, PGM1, EFCAB7, ITGB3BP, 5S_rRNA, ALG6, FOXD3 |
| iHS Nguni  P<0.0001 | 3 | 85100000 | 90400000 | 5.3 | C3H1orf87, HOOK1, FGGY, U6, bta-mir-2285ap, bta-mir-12043, JUN, MYSM1, TACSTD2, OMA1, C8B, C8A, FYB2, PRKAA2, PLPP3 |
|  | 3 | 97800000 | 101700000 | 3.9 | SPATA6, SLC5A9, SKINT1, U6, TRABD2B, 5S_rRNA, FOXD2, FOXE3, CMPK1, STIL, 5S_rRNA, U6, TAL1, PDZK1IP1, CYP4X1, 5S_rRNA, CYP4A11, CYP4A22, CYP4B1, EFCAB14, TEX38, ATPAF1, MOB3C, MKNK1, KNCN, TMEM275, DMBX1, FAAH, NSUN4, UQCRH, LRRC41, RAD54L, POMGNT1, LURAP1, TSPAN1, P3R3URF, U6, PIK3R3, MAST2, IPP, TMEM69, GPBP1L1, CCDC17, NASP, AKR1A1, PRDX1, MMACHC, TESK2, TOE1,MUTYH, HPDL, ZSWIM5, UROD, HECTD3, EIF2B3, PTCH2, U5, BTBD19, DYNLT4, PLK3, BEST4, RPS8, SNORD39, U6, KIF2C, U5, ARMH1, TMEM53, RNF220 |
|  | 3 | 110900000 | 112900000 | 2 | GJB3, GJB4, GJB5, C3H1orf94, CSMD2, HMGB4, U6, ZSCAN20, EFHD1, GIGYF2, KCNJ13, SNORC, NGEF, NEU2, INPP5D |
|  | 4 | 21300000 | 23200000 | 1.9 | ETV1, DGKB |
|  | 4 | 26600000 | 28500000 | 1.9 | HDAC9, TWIST1, FERD3L, POLR1F, TMEM196 |
|  | 4 | 35100000 | 37500000 | 2.4 | SEMA3D, SEMA3A, SEMA3E, PCLO |
|  | 4 | 48200000 | 63000000 | 14.8 | PRKAR2B, HBP1, COG5, GPR22, DUS4L, SLC26A4, CBLL1, SLC26A3, DLD, LAMB1, bta-mir-2418, NRCAM, PNPLA8, NME8, SFRP4, EPDR1, STARD3NL, TARP, CTTNBP2, CFTR, ASZ1, WNT2, ST7, CAPZA2, MET, CAV1, CAV2, TES, TFEC, MDFIC, FOXP2, UBE2I, PPP1R3A, GPR85, BMT2, TMEM168, LSMEM1, IFRD1, ZNF277, DOCK4, IMMP2L, LRRN3, RGS2, DNAJB9, THAP5, GPR141, ELMO1, AOAH, ANLN, KIAA0895, EEPD1, SEPTIN7, HERPUD2, TBX20, DPY19L2, DPY19L1, NPSR1, BMPER, bta-mir-2285bu-2 |
|  | 4 | 66100000 | 68000000 | 1.9 | MTURN, PLEKHA8, FKBP14, SCRN1, WIPF3, PRR15, CHN2, CPVL, TRIL, CREB5, JAZF1 |
|  | 4 | 89200000 | 91100000 | 1.9 | GRM8 |
|  | 4 | 96500000 | 98400000 | 1.9 | CHCHD3, EXOC4, bta-mir-2423, LRGUK, SLC35B4, AKR1B1, AKR1B10, BPGM |
|  | 4 | 104200000 | 106100000 | 1.9 | MRPS33, AGK, DENND11, WEE2, SSBP1, TAS2R3, TAS2R4, PRSS37, OR9A4, OR9A17, OR9A18, OR9A7, CLEC5A, TAS2R38, MGAM, PRSS58, TRBV3-1, TRBV15, TRBV24-1, PRSS2 |
|  | 5 | 47400000 | 50400000 | 3 | GRIP1, HELB, IRAK3, TMBIM4, LLPH, 5S_rRNA, HMGA2, bta-mir-763, MSRB3, LEMD3, WIF1, U6, TBC1D30, GNS, RASSF3, TBK1, bta-mir-2285f-1, XPOT, C5H12orf56, C5H12orf66, SRGAP1, RXYLT1, AVPR1A |
| iHS Nguni  P<0.0001 | 5 | 97600000 | 101300000 | 3.7 | LRP6, BCL2L14, ETV6, TAS2R42, SMIM10L1, TAS2R46, T2R65A, T2R12, BOTA-T2R10B, TAS2R10, T2R10C, TAS2R8, TAS2R7, YBX3, STYK1, MAGOHB, KLRA1, KLRC1, KLRJ1, NKG2A, NKG2C, KLRK1, GABARAPL1, TMEM52B, OLR1, CLEC7A, CLEC1A, CLEC9A, CLEC1B, CLEC12B, CLEC12A, CLEC2A, KLRF2, 7SK, bta-mir-12002a, bta-mir-12002b, KLRF1, CD69, CLECL1, KLRB1, OVOS2, A2M, KLRG1, M6PR, PHC1, A2ML1, bta-mir-2284r, RIMKLB, MFAP5, AICDA, APOBEC1 |
|  | 5 | 105600000 | 107500000 | 1.9 | C5H12orf4, FGF6, FGF23, TIGAR, CCND2, PARP11, CRACR2A, PRMT8, TSPAN11, TSPAN9, TEAD4, TULP3, RHNO1, FOXM1, TEX52, NRIP2, ITFG2, FKBP4, WASHC1, IQSEC3, SLC6A12, SLC6A13, KDM5A, CCDC77, B4GALNT3, SNORA70, NINJ2 |
|  | 6 | 28000000 | 29900000 | 1.9 | PDHA2, UNC5C, BMPR1B |
|  | 6 | 36600000 | 38600000 | 2 | ABCG2, PKD2, SPP1, MEPE, IBSP, LAP3, MED28, FAM184B, NCAPG, DCAF16, LCORL |
|  | 6 | 47800000 | 52400000 | 4.6 | Y_RNA, PCDH7 |
|  | 6 | 77600000 | 79500000 | 1.9 | ADGRL3 |
|  | 7 | 22800000 | 24700000 | 1.9 | FNIP1, bta-mir-12018, 7SK, CDC42SE2, LYRM7, HINT1, CHSY3, MINAR2, ADAMTS19 |
|  | 7 | 49600000 | 51500000 | 1.9 | BRD8, CDC23, KIF20A, GFRA3, CDC25C, SLBP2, FAM53C, bta-mir-2459, KDM3B, REEP2, EGR1, ETF1, HSPA9, SNORD63, CTNNA1, 5S_rRNA, LRRTM2, SIL1, bta-mir-1949, SNORA74, MATR3, PAIP2, SLC23A1, MZB1, PROB1, SPATA24, DNAJC18, ECSCR, SMIM33, STING1, UBE2D2, CXXC5, PSD2, NRG2, PURA, IGIP, CYSTM1, PFDN1 |
|  | 7 | 83800000 | 86600000 | 2.8 | EDIL3 |
|  | 7 | 92200000 | 94800000 | 2.6 | NR2F1, FAM172A, KIAA0825, SLF1, MCTP1, bta-mir-2284z-7, FAM81B, TTC37 |
|  | 9 | 21900000 | 26300000 | 4.4 | IBTK, TPBG, UBE3D, DOP1A, PGM3, RWDD2A, ME1, PRSS35, SNAP91, C9H6orf58, KIAA0408, ECHDC1, RNF146, RSPO3, CENPW, bta-mir-2284o, TRMT11, HINT3, NCOA7, HEY2, HDDC2, bta-mir-2477, TPD52L1, RNF217 |
| iHS Nguni  P<0.0001 | 10 | 25800000 | 27700000 | 1.9 | SUPT16H, RPGRIP1, HNRNPC, OR5AU1, TMEM253, ZNF219, ARHGEF40, RNASE13, TPPP2, NDRG2, SLC39A2, METTL17, RNASE2, BRB, RNASE1, RNASE6, RNASE4, ANG2, OR6S1, RNASE12, RNASE11, RNASE10, PNP, PIP4P1, APEX1, OSGEP, KLHL33, TEP1, PARP2, RNaseP_nuc, RPS29, CCNB1IP1, TTC5, OR11H4, OR11G9, OR11H7, OR11H6, OR11G2, OR11G2C, OR11G7, OR11G2B, OR11J4, OR11J12, OR11J6, OR11K2, OR11K5, OR11G11, OR11H12, OR11H12B, OR11G29, OR4Q3, OR4H12, OR4M1, OR4N2C, OR4N4, OR4N2, OR4K6, OR4K3, OR4K2, OR4K5, OR4K1, OR4K15, OR4Q2, OR4K14, OR4L1, OR4L18, OR4N5, OR4K55B, OR4K35, OR4G21, OR4F69, OR4G10, OR4F67B, OR4G18, OR4K36 |
|  | 10 | 28200000 | 30100000 | 1.9 | OR4F68, OR4F34, LPCAT4, NUTM1, NOP10, SLC12A6, EMC4, KATNBL1, EMC7, CHRM5, AVEN, RYR3, bta-mir-2285as-1, TMCO5B, FMN1, GREM1, SCG5 |
|  | 10 | 31000000 | 32900000 | 1.9 | DPH6, CDIN1, MEIS2, U4 |
|  | 10 | 36000000 | 37900000 | 1.9 | KNSTRN, IVD, BAHD1, CHST14, CCDC32, RPUSD2, KNL1, RAD51, GCHFR, DNAJC17, C10H15orf62, ZFYVE19, PPP1R14D, SPINT1, RHOV, VPS18, DLL4, CHAC1, INO80, EXD1, CHP1, OIP5, NUSAP1, NDUFAF1, RTF1, ITPKA, RPAP1, TYRO3, MGA, bta-mir-2285cr-1 ,MAPKBP1, JMJD7, PLA2G4B, SPTBN5, EHD4, PLA2G4E, PLA2G4D, PLA2G4F, VPS39, TMEM87A, GANC, CAPN3, ZNF106, SNAP23, LRRC57, HAUS2 |
|  | 10 | 46000000 | 48200000 | 2.2 | DAPK2, HERC1, FBXL22, USP3, CA12, APH1B, RAB8B, RPS27L, LACTB, TPM1, TLN2, bta-mir-190a, C2CD4B, VPS13C |
|  | 10 | 48600000 | 50500000 | 1.9 | RORA, ICE2, ANXA2, FOXB1 |
|  | 10 | 50800000 | 52700000 | 1.9 | FAM81A, MYO1E, CCNB2, RNF111, U7, SLTM, MINDY2, ADAM10, LIPC, AQP9, ALDH1A2, POLR2M, MYZAP |
|  | 10 | 54300000 | 56700000 | 2.4 | RFX7, NEDD4, PRTG, PYGO1, DNAAF4, C15orf65, CCPG1, bta-mir-628, PIGB, RAB27A, RSL24D1, UNC13C, WDR72 |
|  | 10 | 82000000 | 83900000 | 1.9 | SYNJ2BP, COX16, ADAM21, MED6, ADAM20, TTC9, MAP3K9, PCNX1, SIPA1L1, Metazoa_SRP, RGS6 |
|  | 11 | 8000000 | 11700000 | 3.7 | POU3F3, MRPS9, GPR45, TGFBRAP1, C11H2orf49, FHL2, TACR1, POLE4, U6, HK2, SEMA4F, M1AP, DOK1, LOXL3, HTRA2, AUP1, DQX1, TLX2, PCGF1, LBX2, CCDC142, MRPL53, MOGS, WBP1, INO80B, RTKN, WDR54, C11H2orf81, MGC152281, DCTN1, SLC4A5, MTHFD2, MOB1A, BOLA3, TET3, DGUOK, ACTG2, STAMBP, C11H2orf78, DUSP11, bta-mir-2295, TPRKB, ALMS1, EGR4, FBXO41, CCT7, PRADC1, SMYD5, NOTO, RAB11FIP5, bta-mir-2294, SFXN5, EMX1, SPR, EXOC6B |
| iHS Nguni  P<0.0001 | 11 | 18400000 | 21500000 | 3.1 | CRIM1, FEZ2, VIT, STRN, HEATR5B, GPATCH11, EIF2AK2, SULT6B1, CEBPZ, NDUFAF7, PRKD3, QPCT, CDC42EP3, RMDN2, CYP1B1, ATL2, HNRNPLL, GALM, SRSF7, GEMIN6, DHX57, MORN2, ARHGEF33, SOS1, bta-mir-2284z-2, CDKL4 |
|  | 11 | 26000000 | 27900000 | 1.9 | DYNC2LI1, ABCG5, ABCG8, LRPPRC, PPM1B, SLC3A1, PREPL, CAMKMT, SIX3, SIX2, SRBD1 |
|  | 11 | 65100000 | 67200000 | 2.1 | ETAA1, C1D, WDR92, PNO1, PPP3R1, CNRIP1, PLEK, FBXO48, APLF, PROKR1, ARHGAP25 |
|  | 11 | 84300000 | 86200000 | 1.9 | TRIB2, LPIN1 |
|  | 11 | 91500000 | 93400000 | 1.9 | GGTA1, DAB2IP, NDUFA8, MORN5, LHX6, RBM18, MRRF, PTGS1, bta-mir-10175, OR1L12, OR1L8H, OR1L8, OR1L8C, OR1L8E, OR1L20, OR1L8D |
|  | 12 | 17100000 | 20900000 | 3.8 | SUCLA2, NUDT15, MED4, ITM2B, RB1, LPAR6, RCBTB2, CYSLTR2, FNDC3A, MLNR, CDADC1, CAB39L, SETDB2, PHF11, RCBTB1, ARL11, EBPL, KPNA3, SPRYD7, TRIM13, KCNRG, bta-mir-16a, DLEU7, bta-mir-10177, RNASEH2B, FAM124A, SERPINE3, INTS6 |
|  | 12 | 23100000 | 26700000 | 3.6 | NHLRC3, PROSER1, STOML3, FREM2, UFM1, TRPC4, POSTN, SUPT20H, EXOSC8, ALG5, SMAD9, RFXAP, SERTM1, CCNA1, SPART, DCLK1, NBEA, MAB21L1 |
|  | 12 | 27200000 | 35700000 | 8.5 | RFC3, STARD13, KL, PDS5B, N4BP2L1, BRCA2, ZAR1L, FRY, RXFP2, bta-mir-2299, B3GLCT, HSPH1, TEX26, MEDAG, ALOX5AP, USPL1, HMGB1, KATNAL1, UBL3, SLC7A1, MTUS2, POMP, FLT1, bta-mir-2300a, PAN3, FLT3, bta-mir-2285o-2, URAD, CDX2, PDX1, GSX1, POLR1D, LNX2, MTIF3, GTF3A, RASL11A, RPL21, USP12, GPR12, WASF3, CDK8, RNF6, SHISA2, ATP8A2, NUP58, MTMR6, AMER2, C1QTNF9, MIPEP, TNFRSF19, SACS, SGCG, FGF9, MICU2, ZDHHC20, MRPL57, SKA3, SAP18, LATS2 |
|  | 12 | 38800000 | 41300000 | 2.5 | PCDH9 |
|  | 12 | 48400000 | 54300000 | 5.9 | KLF12, TBC1D4, COMMD6, UCHL3, LMO7, bta-mir-2285ab, KCTD12, ACOD1, CLN5, FBXL3, MYCBP2, SCEL, SLAIN1, bta-mir-2284s, EDNRB, POU4F1, OBI1 |
| iHS Nguni  P<0.0001 | 12 | 56900000 | 58800000 | 1.9 | bta-mir-1256, SLITRK1 |
|  | 12 | 62600000 | 65700000 | 3.1 | bta-mir-17, bta-mir-19a |
|  | 12 | 68600000 | 71200000 | 2.6 | DCT, TGDS, GPR180, SOX21 |
|  | 12 | 77000000 | 80200000 | 3.2 | PCCA, GGACT, TMTC4, NALCN, ITGBL1, FGF14, TPP2, METTL21C, TEX30, POGLUT2, BIVM, ERCC5, METTL21E, SLC10A2 |
|  | 13 | 19200000 | 21100000 | 1.9 | NRP1, ITGB1, ARL5B |
|  | 13 | 28400000 | 30300000 | 1.9 | FRMD4A, FAM107B, CDNF, HSPA14, SUV39H2, DCLRE1C, MEIG1, OLAH, ACBD7, RPP38, NMT2, FAM171A1, ITGA8 |
|  | 13 | 37400000 | 39300000 | 1.9 | PCSK2, BFSP1, DSTN, RRBP1, BANF2 ,SNX5, bta-mir-12034, MGME1, OVOL2, PET117, KAT14, ZNF133, DZANK1, POLR3F, RBBP9, SEC23B, SMIM26, DTD1, SCP2D1, SLC24A3, bta-mir-11973 |
|  | 13 | 43700000 | 45600000 | 1.9 | 20ALPHA-HSD, AKR1E2, KLF6, bta-mir-12028, PITRM1, PFKP |
|  | 13 | 52800000 | 54700000 | 1.9 | TGM3, STK35, PDYN, SIRPA, SIRPB1, GINS1, PCMTD2, MYT1, NPBWR2, OPRL1, LKAAEAR1, RGS19, TCEA2, SOX18, PRPF6, SAMD10, ZNF512B, UCKL1, bta-mir-1388, DNAJC5, TPD52L2, ABHD16B, ZBTB46, SLC2A4RG, ZGPAT, ARFRP1, TNFRSF6B, RTEL1 ,STMN3, GMEB2, FNDC11, SRMS, PTK6, PPDPF, EEF1A2, KCNQ2, CHRNA4, COL20A1, ARFGAP1, BIRC7, YTHDF1, bta-mir-124b, BHLHE23, SLC17A9, GID8, DIDO1, TCFL5, COL9A3, OGFR, MRGBP, NTSR1, SLCO4A1 |
|  | 13 | 55300000 | 58500000 | 3.2 | CDH4, CDH26, FAM217B, PPP1R3D, SYCP2, PHACTR3, bta-mir-2285bz, EDN3, ZNF831, PRELID3B, ATP5F1E, TUBB1, CTSZ, NELFCD, GNAS, bta-mir-6123, NPEPL1, STX16, APCDD1L, VAPB, RAB22A, C13H20orf85, PMEPA1 |
|  | 13 | 62000000 | 66200000 | 4.2 | COMMD7, DNMT3B, MAPRE1, EFCAB8, SUN5, BPIFB2, BPIFB6, BPIFB3, BPIFB4, BPIFA2A, BPIFA2C, BPIFA2B, BPIFA3, BPIFA1, BPIFB1, BPIFB5, CDK5RAP1, SNTA1, NECAB3, C13H20orf144, E2F1, PXMP4 ,ZNF341, CHMP4B, RALY, EIF2S2, ASIP, AHCY, ITCH, DYNLRB1, MAP1LC3A, PIGU, TP53INP2, NCOA6, GGT7, ACSS2, GSS, MYH7B, bta-mir-499, TRPC4AP, EDEM2, PROCR, MMP24, EIF6, FAM83C, UQCC1, GDF5, CEP250, ERGIC3, SPAG4, CPNE1, RBM12, NFS1, ROMO1, RBM39, PHF20, SCAND1, CNBD2, EPB41L1, AAR2, DLGAP4, MYL9, TGIF2, RAB5IF, SLA2, NDRG3, DSN1, SOGA1, TLDC2, SAMHD1, RBL1, MROH8, RPN2 |
| iHS Nguni  P<0.0001 | 14 | 23300000 | 27600000 | 4.3 | MOS, PLAG1, CHCHD7, SDR16C5, SDR16C6, PENK, BPNT2, FAM110B, UBXN2B, CYP7A1, U1, SDCBP, NSMAF, TOX, CA8, RAB2A, CHD7, CLVS1, ASPH |
|  | 14 | 37300000 | 39500000 | 2.2 | JPH1, GDAP1, PI15, CRISPLD1, HNF4G |
|  | 14 | 55900000 | 57800000 | 1.9 | EMC2, EIF3E, RSPO2, ANGPT1 |
|  | 14 | 60600000 | 63200000 | 2.6 | RIMS2, DCAF13, SLC25A32, CTHRC1, FZD6, BAALC, ATP6V1C1, AZIN1, KLF10, ODF1, UBR5, RRM2B, NCALD, GRHL2, ZNF706 |
|  | 15 | 31100000 | 33500000 | 2.4 | GRIK4, bta-mir-2284j ,TBCEL, TECTA, SC5D, SORL1, bta-mir-125b-1, bta-let-7a-2, bta-mir-100, UBASH3B, CRTAM |
|  | 15 | 36000000 | 38300000 | 2.3 | SOX6, INSC, CALCB, CALCA, CYP2R1, PDE3B, PSMA1, COPB1 |
|  | 15 | 41200000 | 43100000 | 1.9 | GALNT18, ZBED5, EIF4G2, CTR9, IRAG1, LYVE1, RNF141, bta-mir-2315, AMPD3, ADM, bta-mir-6518, SBF2, SWAP70, WEE1 |
|  | 15 | 45000000 | 46900000 | 1.9 | OVCH2, CYB5R2, PPFIBP2, OLFML1, SYT9, RBMXL2, NLRP14, ZNF214, ZNF215, OR2D3, OR2D2, OR10A4, OR10A5, OR10A5L, OR10A5G, OR6A2, OR6B18, OR6B17, OR2D4, OR2D3G, OR2AG1E, OR2AG1G, OR2AG1, OR2AG2, MRPL17, DCHS1, TPP1, TAF10, ILK, RRP8, TIMM10B, ARFIP2, TRIM3, HPX, APBB1, SMPD1, bta-mir-2316, CAVIN3, CCKBR, CNGA4, FAM160A2, C15H11orf42, OR52W1, OR52B2, OR56B42 |
|  | 15 | 51900000 | 53800000 | 1.9 | CLPB, PDE2A, bta-mir-139, ARAP1, STARD10, ATG16L2, FCHSD2, P2RY2, P2RY6, ARHGEF17, RELT, FAM168A, PLEKHB1, RAB6A, MRPL48, CHCHD8, PAAF1, DNAJB13, UCP2, UCP3, C2CD3, PPME1, P4HA3 |
|  | 15 | 54700000 | 56600000 | 1.9 | PGM2L1, GDPD5, SERPINH1, MAP6, MOGAT2, UVRAG, WNT11, THAP12, EMSY, LRRC32, TSKU, ACER3, B3GNT6, CAPN5, MYO7A |
|  | 16 | 23800000 | 25700000 | 1.9 | MARK1, C16H1orf115, MTARC2, MTARC1, HLX, DUSP10, U8 |
|  | 16 | 29600000 | 33300000 | 3.7 | STUM, ITPKB, PSEN2, COQ8A, CDC42BPA, AHCTF1, SCCPDH, H3-3D, CNST, TFB2M, SMYD3, KIF26B, EFCAB2, HNRNPU, COX20, DESI2, ADSS2, C16H1orf100, ZBTB18 |
|  | 16 | 36100000 | 38000000 | 1.9 | XCL1, DPT, ATP1B1, SNORA66, NME7, BLZF1, CCDC181, SLC19A2, F5, SELP, SELL, SELE, METTL18, SCYL3, KIFAP3, METTL11B |
|  | 17 | 43200000 | 48000000 | 4.8 | CTSO, TDO2,ASIC5, GUCY1B1, GUCY1A1, ZNF605, ZNF26, ZNF84, ZNF140, ZNF891, ZNF10, ZNF268, MBD3L1, ANHX, CHFR, GOLGA3, ANKLE2, PGAM5, PXMP2, POLE, P2RX2, LRCOL1, FBRSL1, GALNT9, NOC4L, DDX51, EP400, bta-mir-6520, PUS1, ULK1, MMP17, SFSWAP, bta-mir-2285af-2, ADGRD1, RAN, STX2, RIMBP2, PIWIL1, FZD10 ,TMEM132D |
| iHS Nguni  P<0.0001 | 17 | 49500000 | 51400000 | 1.9 | TMEM132B, AACS, bta-mir-10166, BRI3BP, DHX37, SNORA71, UBC, SCARB1, NCOR2 |
|  | 17 | 52100000 | 54000000 | 1.9 | RILPL1, SNRNP35, RILPL2, KMT5A, SBNO1, CDK2AP1, C17H12orf65, MPHOSPH9, PITPNM2, ARL6IP4, OGFOD2, ABCB9, VPS37B, HIP1R, CCDC62, DENR, SNORA70, HCAR1, KNTC1, RSRC2, ZCCHC8, CLIP1, VPS33A, DIABLO, B3GNT4, LRRC43, MLXIP, BCL7A, CFAP251, PSMD9, HPD, SETD1B, RHOF, TMEM120B, MORN3, ORAI1, KDM2B, RNF34, ANAPC5, CAMKK2, P2RX4, P2RX7 |
|  | 17 | 60600000 | 62500000 | 1.9 | RBM19, LHX5, SDSL, SDS, PLBD2, DTX1, RASAL1, CFAP73, DDX54, RITA1, IQCD, TPCN1, SLC8B1, OAS2, OAS1Y, OAS1X, OAS1Z, RPH3A, PTPN11, RPL6, HECTD4, TRAFD1, NAA25, ERP29, TMEM116, MAPKAPK5, ALDH2, BICDL1, RAB35 |
|  | 18 | 4000000 | 5900000 | 1.9 | MON1B, ADAMTS18, NUDT7, VAT1L, CLEC3A, WWOX, bta-mir-2285al |
|  | 18 | 19300000 | 21200000 | 1.9 | SALL1, TOX3 |
|  | 18 | 22400000 | 24300000 | 1.9 | FTO, IRX3, IRX5, IRX6, MMP2, LPCAT2, SLC6A2, CES1, MT1A, MT1E, MT2A, MT3, MT4, BBS2, OGFOD1, NUDT21, AMFR, GNAO1 |
|  | 18 | 42000000 | 43900000 | 1.9 | bta-mir-2899, ZNF507, DPY19L3, PDCD5, RGS9BP, NUDT19, TDRD12, SLC7A9, CEP89, FAAP24, RHPN2, GPATCH1, WDR88, LRP3, SLC7A10, CEBPG, PEPD |
|  | 18 | 45500000 | 47400000 | 1.9 | ZNF181, ZNF599, ZNF792, GRAMD1A, SCN1B, HPN, LGI4, FXYD1, FXYD7, FXYD5, FAM187B, LSR, USF2, HAMP, MAG, CD22, FFAR1, FFAR3, FFAR2, KRTDAP, DMKN, SBSN, GAPDHS, TMEM147, ATP4A, HAUS5, RBM42, ETV2, COX6B1, UPK1A, ZBTB32, KMT2B, IGFLR1, U2AF1L4, PSENEN, LIN37, HSPB6, PROSER3, ARHGAP33, PRODH2, NPHS1, KIRREL2, APLP1, TYROBP, NFKBID, HCST, LRFN3, SDHAF1, SYNE4, ALKBH6, CLIP3, THAP8, OVOL3, POLR2I, TBCB, CAPNS1, COX7A1, ZNF565, ZNF567, ZNF382, ZNF529, ZNF566, ZFP14, ZNF568, ZNF793, ZNF383, ZNF527 |
| iHS Nguni  P<0.0001 | 18 | 47600000 | 49500000 | 1.9 | ZFP30, SIPA1L3, DPF1, PPP1R14A, SPINT2, C18H19orf33, YIF1B, KCNK6, CATSPERG, PSMD8, GGN, SPRED3, FAM98C, RASGRP4, RYR1, MAP4K1, EIF3K, ACTN4, CAPN12, LGALS4, ECH1, HNRNPL, RINL, SIRT2, NFKBIB, CCER2, SARS2, MRPS12, FBXO17, ACP7, PAK4, NCCRP1, SYCN, IFNL3, LRFN1, GMFG, SAMD4B, PAF1, MED29, ZFP36, PLEKHG2, RPS16, IL-15L, SUPT5H, TIMM50, DLL3, SELENOV, EID2, DYRK1B, FBL, PSMC4 |
|  | 18 | 51400000 | 53300000 | 1.9 | CXCL17, CD177, TEX101, BSP3, BSP5, BSP1, LYPD3, PHLDB3, ETHE1, ZNF575, XRCC1, PINLYP, IRGQ, ZNF576, ZNF428, SRRM5, CADM4, PLAUR, IRGC, SMG9, KCNN4, LYPD5, ZNF283, ZNF404, ZNF45, ZNF226, ZNF234, ZNF227, ZNF235, ZNF112, ZNF180, IGSF23, PVR, CEACAM19, CEACAM16, BCL3, CBLC, BCAM, NECTIN2, TOMM40, APOE, APOC4, APOC2, CLPTM1, RELB, CLASRP, ZNF296, GEMIN7, PPP1R37, NKPD1, TRAPPC6A, BLOC1S3, EXOC3L2, MARK4, CKM, KLC3, ERCC2, PPP1R13L, POLR1G, ERCC1, FOSB, RTN2, PPM1N, VASP, OPA3, GPR4, EML2, bta-mir-330, GIPR, SNRPD2, QPCTL, FBXO46 |
|  | 19 | 28300000 | 31800000 | 3.5 | PIK3R6, PIK3R5, bta-mir-2284aa-3, NTN1, STX8, CFAP52, USP43, DHRS7C, GLP2R, RCVRN, GAS7, MYH13, MYH8, MYH4, MYH1, MYH2, MYH3, SCO1, ADPRM, TMEM220, PIRT, DNAH9, ZNF18, MAP2K4, bta-mir-744, MYOCD, ARHGAP44, ELAC2, bta-mir-2339, HS3ST3A1 |
|  | 19 | 41200000 | 46600000 | 5.4 | KRTAP3-1, KRTAP1-1, KRTAP4-7, KRTAP9-1, KRT33A, KRT33B, KRT34, KRT31, KRT37, KRT32, KRT35, KRT36, KRT15, KRT19, KRT9, KRT14, KRT16, KRT17, KRT42, EIF1, GAST, HAP1, JUP, P3H4, FKBP10, NT5C3B, KLHL10, KLHL11, ACLY, ODAD4, CNP, DNAJC7, NKIRAS2, ZNF385C, C19H17orf113, DHX58, KAT2A, RAB5C, HSPB9, KCNH4, HCRT, GHDC, STAT5B, STAT5A, STAT3, CAVIN1, ATP6V0A1, NAGLU, HSD17B1, COASY, MLX, PSMC3IP, RETREG3, TUBG1, TUBG2, PLEKHH3, CCR10, CNTNAP1, EZH1, RAMP2, VPS25, WNK4, COA3, CNTD1, BECN1, PSME3, AOC2 |
|  | 23 | 15900000 | 18100000 | 2.2 | GUCA1B, MRPS10, TRERF1, UBR2, PRPH2, TBCC, BICRAL, RPL7L1, PTCRA, CNPY3, GNMT, PEX6, PPP2R5D, MEA1, KLHDC3, RRP36, CUL7, MRPL2, KLC4, PTK7, SRF, CUL9, DNPH1, TTBK1, SLC22A7, CRIP3, ZNF318, ABCC10, DLK2, TJAP1, LRRC73, YIPF3, POLR1C, XPO5, POLH, GTPBP2, MAD2L1BP, RSPH9, MRPS18A, VEGFA, TMEM63B, CAPN11, MYMX, SLC29A1, HSP90AB1, SLC35B2, NFKBIE, TMEM151B, AARS2, SPATS1, CDC5L |
| iHS Nguni  P<0.0001 | 23 | 21300000 | 25000000 | 3.7 | OR9G1, OR5M10C, MMUT, CENPQ, GLYATL3, C23H6orf141, RHAG, CRISP2, CRISP3, CRISP1, DEFB114, DEFB110, TFAP2D, TFAP2B, PKHD1, bta-mir-206, IL17A, IL17F, MCM3, PAQR8, EFHC1, TRAM2 |
|  | 23 | 26000000 | 28400000 | 2.4 | NOTCH4, GPSM3, PBX2, AGER, RNF5, AGPAT1, EGFL8, PPT2, PRRT1, FKBPL, ATF6B, TNXB, CYP21, C4A, STK19, DXO, SKIV2L, NELFE, CFB, C2, ZBTB12, EHMT2, SLC44A4, NEU1, HSPA1A, HSPA1L, LSM2, VARS1, VWA7, SAPCD1, MSH5, CLIC1, DDAH2, MPIG6B, LY6G6C, LY6G6E, LY6G6F, ABHD16A, LY6G5C, LY6G5B, CSNK2B, GPANK1, C23H6orf47, APOM, BAG6, PRRC2A, AIF1, NCR3, LTB, TNF, LTA, NFKBIL1, ATP6V1G2, DDX39B, MCCD1, BoLA, bta-mir-10167, MIC1, MIC2, POU5F1, TCF19, CCHCR1, PSORS1C2, CDSN, C23H6orf15, SFTA2, VARS2, GTF2H4, DDR1, IER3, FLOT1, TUBB, MDC1, NRM, PPP1R18, DHX16, C23H6orf136, ATAT1, MRPS18B, PPP1R10, ABCF1, bta-mir-2378, bta-mir-877 |
|  | 23 | 38400000 | 40300000 | 1.9 | RNF144B, DEK, KDM1B, TPMT, NHLRC1, KIF13A, NUP153, FAM8A1, CAP2, RBM24, bta-mir-2325b, STMND1 |
|  | 23 | 46000000 | 47900000 | 1.9 | SLC35B3, EEF1E1, BLOC1S5, TXNDC5, BMP6, SNRNP48, DSP |
|  | 24 | 26800000 | 28700000 | 1.9 | CDH2 |
|  | 24 | 51400000 | 53800000 | 2.4 | DCC, MBD2, POLI, STARD6, C24H18orf54, CUPIN1 |
|  | 24 | 54900000 | 57400000 | 2.5 | TCF4, TXNL1, WDR7, ST8SIA3, ONECUT2, FECH, NARS1, ATP8B1, NEDD4L |
|  | 25 | 30600000 | 33600000 | 3 | SNORD14, 5_8S_rRNA, RCC1L, GTF2IRD2, NCF1, GTF2I, GTF2IRD1, bta-mir-2387, CLIP2, RFC2, LAT2, EIF4H, LIMK1, ELN, TMEM270, METTL27, CLDN4, CLDN3, ABHD11, STX1A, BUD23, DNAJC30, VPS37D |
|  | 26 | 6600000 | 8500000 | 1.9 | DKK1, PRKG1, CSTF2T, A1CF |
|  | 26 | 8900000 | 10800000 | 1.9 | SGMS1, MINPP1, PAPSS2, ATAD1, PTEN, RNLS, LIPJ, LIPF, LIPK, LIPN, LIPM, ANKRD22, STAMBPL1, ACTA2, FAS |
|  | 26 | 28900000 | 30800000 | 1.9 | bta-mir-2285k-4, XPNPEP1, ADD3, MXI1 |
|  | 27 | 7200000 | 9100000 | 1.9 | GPM6A, WDR17, SPATA4, ASB5, SPCS3, VEGFC, NEIL3, AGA |
|  | 27 | 13200000 | 15600000 | 2.4 | TENM3, DCTD, WWC2, CDKN2AIP, ING2, RWDD4, TRAPPC11, STOX2, ENPP6, IRF2, CASP3, PRIMPOL, ACSL1, CENPU, HELT, SLC25A4, CFAP97, SNX25 |
|  | 27 | 16600000 | 19600000 | 3 | U3, ZFP42, TRIML2, TRIML1, FRG1, ASAH1, PCM1, bta-mir-2398, FGL1, bta-mir-2285ai, MTUS1 |
| iHS Nguni  P<0.0001 | 29 | 39600000 | 42000000 | 2.4 | PAG8, PGA5, VWCE, DDB1, TKFC, CYB561A3, TMEM138, TMEM216, CPSF7, SDHAF2, bta-mir-2405, PPP1R32, LRRC10B, SYT7, bta-mir-2885, DAGLA ,MYRF, TMEM258, FEN1, FADS1, FADS2, FADS3, RAB3IL1, BEST1, FTH1, INCENP, SCGB1D, SCGB2A2, ASRGL1, SCGB1A1, AHNAK, EEF1G, TUT1, MTA2, EML3, ROM1, B3GAT3, GANAB, INTS5, C29H11orf98, CSKMT, UQCC3, UBXN1, LRRN4CL, BSCL2, GNG3, HNRNPUL2, TTC9C, ZBTB3, POLR2G, TAF6L, TMEM179B, TMEM223, NXF1, STX5, WDR74, U2, SNORD22, SNORD31, SNORD30, SNORD29, SNORD28, SNORD27, SNORD26, SNORD25, SLC3A2, CHRM1, SLC22A6, SLC22A8, SLC22A10, SLC22A9, PLAAT5, LGALS12, PLA2G16 |

*Length of candidate region is measured in mega base pair (Mb), where 1Mb = 1 000 000 bases.
